# Evaluating Cross-Applicability of Weed Detection Models Across Different Crops in Similar Production Environments

**DOI:** 10.3389/fpls.2022.837726

**Published:** 2022-04-28

**Authors:** Bishwa B. Sapkota, Chengsong Hu, Muthukumar V. Bagavathiannan

**Affiliations:** ^1^Department of Soil and Crop Sciences, Texas A&M University, College Station, TX, United States; ^2^Department of Biological and Agricultural Engineering, College Station, TX, United States

**Keywords:** deep learning, CNNs, digital technologies, precision weed control, site-specific weed management, precision agriculture

## Abstract

Convolutional neural networks (CNNs) have revolutionized the weed detection process with tremendous improvements in precision and accuracy. However, training these models is time-consuming and computationally demanding; thus, training weed detection models for every crop-weed environment may not be feasible. It is imperative to evaluate how a CNN-based weed detection model trained for a specific crop may perform in other crops. In this study, a CNN model was trained to detect morningglories and grasses in cotton. Assessments were made to gauge the potential of the very model in detecting the same weed species in soybean and corn under two levels of detection complexity (levels 1 and 2). Two popular object detection frameworks, YOLOv4 and Faster R-CNN, were trained to detect weeds under two schemes: Detect_Weed (detecting at weed/crop level) and Detect_Species (detecting at weed species level). In addition, the main cotton dataset was supplemented with different amounts of non-cotton crop images to see if cross-crop applicability can be improved. Both frameworks achieved reasonably high accuracy levels for the cotton test datasets under both schemes (Average Precision-AP: 0.83–0.88 and Mean Average Precision-mAP: 0.65–0.79). The same models performed differently over other crops under both frameworks (AP: 0.33–0.83 and mAP: 0.40–0.85). In particular, relatively higher accuracies were observed for soybean than for corn, and also for complexity level 1 than for level 2. Significant improvements in cross-crop applicability were further observed when additional corn and soybean images were added to the model training. These findings provide valuable insights into improving global applicability of weed detection models.

## Introduction

Weeds are major pests in agricultural landscapes that can cause serious crop yield losses (Buchanan and Burns, 1970; Nave and Wax, 1971). A multi-tactic approach to weed management has become vital to thwart herbicide-resistant weed issues in global cropping systems (Bagavathiannan and Davis, 2018), and site-specificity is expected to improve control outcomes and conserve management inputs (Beckie et al., 2019). Injudicious use of agrochemicals has been linked to negative effects on non-target organisms and the broader environment (Liu and Bruch, 2020). Under the conventional broadcast approach, weed control tactics are applied without any regard to weed distribution and densities in the field. Weeds that escape the pre-emergent herbicides or mechanical tillage typically occur sparsely across the field. In such situations, weed control tactics can instead be strictly focused on areas of weed occurrence to save resources (Berge et al., 2012). In recent years, great efforts have been placed for developing and utilizing ground robots (Kargar and Shirzadifar, 2013; Aravind et al., 2015; Sujaritha et al., 2017; Lottes et al., 2019) and unmanned aerial systems (UAS) for site-specific weed control (Ahmad et al., 2020; Martin et al., 2020).

The precision weed control platforms ranging from ground robots to UAS-based selective spraying systems depend greatly on weed detection using computer vision techniques (Liu and Bruch, 2020; Machleb et al., 2020). The overall approach is to detect weeds in digital images and use the local or real world coordinates of the detected objects for site-specific control operations (López-Granados, 2011). In addition to weed control, these techniques offer tremendous opportunities for advancing weed ecology and biology research. Several image-based weed detection techniques have been proposed and implemented. Based on developments made so far, these techniques can be broadly categorized into two main groups: (1) traditional segmentation and machine learning-based techniques (Wu et al., 2011; Ahmed et al., 2012; Rumpf et al., 2012; García-Santillán and Pajares, 2018; Sabzi et al., 2018; Sapkota et al., 2020) and (2) advanced computer vision using convolution neural networks (CNNs; Adhikari et al., 2019; Ma et al., 2019; Sharpe et al., 2020; Hu et al., 2021; Xie et al., 2021).

The CNNs are a specialized type of neural networks that are designed to extract multi-scale features and merge semantically similar features for better prediction and/or detection (LeCun et al., 2015). The use of CNNs in weed detection tasks has gained great attention lately due to their ability to learn complex features through dense and rigorous feature representations (e.g., Xie et al., 2021). The attention has been fostered by the transfer learning concept in CNN that allows the sharing of common model weights from pre-trained models across different tasks (Abdalla et al., 2019; Fawakherji et al., 2019). The CNN-based object detection models have witnessed remarkable breakthroughs recently, and some of the detectors that have been widely used today for various detection tasks are Fast R-CNN (Girshick, 2015), Single-Shot Detector (Liu et al., 2016), Faster R-CNN (Ren et al., 2017), You Only Look Once (YOLO; Redmon et al., 2016), YOLOv3 (Redmon and Farhadi, 2018), YOLOv4 (Bochkovskiy et al., 2020), and more recently YOLOv5.

With respect to weed detection, different CNN-based detection frameworks have been successfully applied for various tasks. Gao et al. (2020) used YOLOv3 and Tiny YOLO models for detection of *Convolvulus sepium* (hedge bindweed) in *Beta vulgaris* (sugar beets) using field-collected and synthetic images. Using the same models, Jiang et al. (2020) also detected both grass and broadleaf weed species, including *Cirsium setosum*, *Descurainia sophia*, *Euphorbia helioscopia*, *Veronica didyma*, and *Avena fatua* in UAS-based Red-Green-Blue (RGB) imageries. Sharpe et al. (2020) detected goosegrass [*Eleusine indica* (L.) Gaertn.] in handheld digital camera-derived images obtained from two different horticultural crops, strawberry, and tomato, using YOLOv3-tiny model. Using YOLOv3, Partel et al. (2019) detected *Portulaca* spp. in pepper (*Capsicum annum*) for a precision spraying system. Yu et al. (2019) employed DetectNet to detect dandelion (*Taraxacum officinale*), ground ivy (*Glechoma hederacea*), and spotted spurge (*Euphorbia maculata*) in perennial ryegrass. Hu et al. (2021) tested Faster R-CNN, DeepLabv3, and Mask R-CNN for broadleaf and grass weed detection in cotton (*Gossypium hirsutum*) and soybean (*Glycine max*) using UAS-borne high-resolution images.

Cross-applicability of the deep learning models for weed detection across different crops is vital for two important reasons. First, several weed species continuously occur in the rotational crops in a given production field [e.g., *Amaranthus palmeri* (Palmer amaranth) occurring in both soybean and corn (*Zea mays*) grown in rotation], and computer vision models should be able to detect these weeds in all crops in the production system. Second, it is likely that the dominant weed species might be similar across production fields within a locality, and the ability to use these models across multiple production fields might be beneficial from efficiency and economic standpoint. This is because CNN models usually require a large set of annotated training images for better performance (Oquab et al., 2014; Gao et al., 2020), which can be difficult to obtain at times.

When only the weeds are annotated in the images and trained for detection, the model considers the crops in the same images as part of the background during the training process. Therefore, during inference, different crops may mimic different backgrounds for the same trained weeds in the images. It is therefore unclear how changes in the background (crop species in our case) may affect weed detection accuracies for different object detection frameworks under different detection scenarios. To the best of our knowledge, no study has looked at the cross-applicability of weed detection models across three of the most popular row crops in the United States: cotton, corn, and soybean. Such an investigation can further advance our understanding of weed detection models and help unleash their full potential.

The main goal of the study was to build a model for weed detection in cotton and investigate the use of the same model for detection of the same weed spectrum in corn and soybean. This study has two specific objectives: (1) build and evaluate models for weed detection in cotton under two weed detection schemes (detection of weeds at the meta-level, and detection at the individual weed species level), and (2) evaluate the performance of the cotton-based model on corn and soybean at different levels of detection complexity.

## Materials and Methods

### Study Area and Experimental Setup

**Figure 1:**
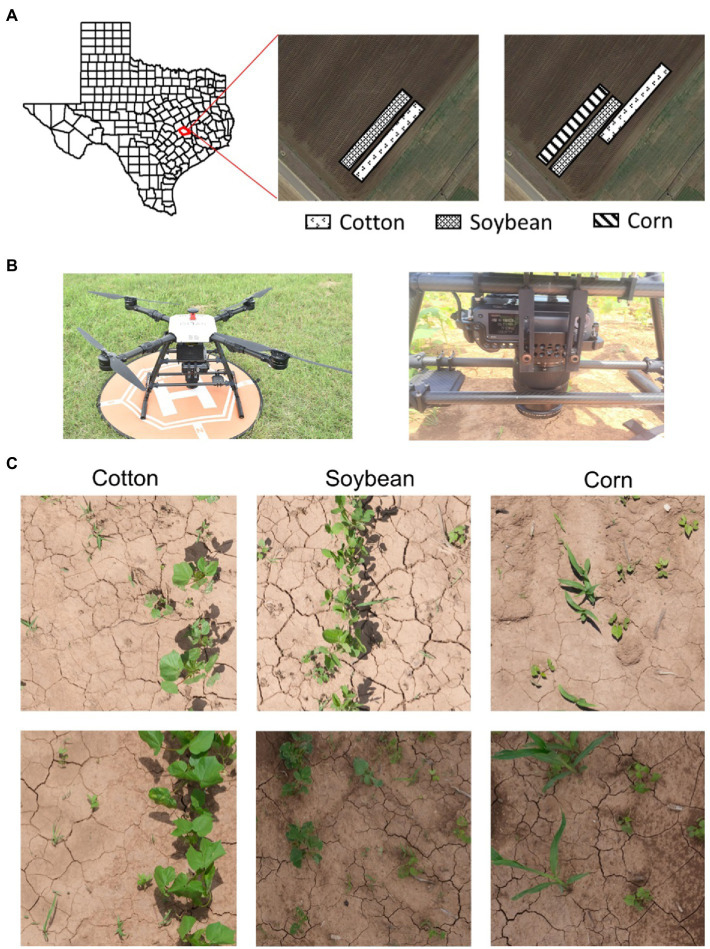
**(A)** Study area (Texas A&M AgriLife Research Farm, Burleson County, TX, United States) and field setup for the 2 experimental years; **(B)** a multi-copter drone (Hylio Inc., Houston, TX, United States) attached with Fujifilm GFX100 (100 MP) camera; and **(C)** image datasets (top and bottom rows) collected under two different environmental conditions for cotton, soybean, and corn.

The study was conducted during the summers of 2020 and 2021 at the Texas A&M AgriLife Research farm (30°32′15″N, 96°25′35″W; elevation: 60 m). The location is characterized by a sub-tropical climate, with an average monthly maximum and minimum air temperatures during the study period (May–June) of 32.3 and 21.3°C, respectively. Glyphosate-resistant (Roundup Ready®) cotton and glufosinate-resistant (Liberty Link®) soybean were planted in two separate strips (Figure 1) adjacent to each other on May 1, 2020, and April 20, 2021, at the seeding rates of 100,000 and 312,500 per hectare, respectively. Each crop was planted using a 4-row seed drill (row spacing: 1 m), with strip sizes of 16 m × 30 m (2020) or 8 m × 40 m (2021). In 2021, corn (Roundup Ready®) was also planted (8 m × 40 m) adjacent to these crops at a seeding rate of 150,000 ha^−1^. The fields were irrigated and fertilized as needed. The crops were grown following the recommended production practices for the region.

In this study, weeds that escaped preemergence and early postemergence herbicide applications were targeted for building and testing models. To this effect, postemergence applications of appropriate herbicides were made in all three crops following standard application procedures, resulting in random escapes at sufficient densities for imaging (Table 1). The dominant weed species in the study area were a mix of morningglories (*Ipomoea* spp.) that composed of tall morningglory (*Ipomoea purpurea*) and ivyleaf morningglory (*Ipomoea hederacea*), Texas millet (*Urochloa texana*), and johnsongrass (*Sorghum halepense*). Some other weed species occurred at low frequencies, including Palmer amaranth (*Amaranthus palmeri*), prostrate spurge (*Euphorbia humistrata*), and browntop panicum (*Panicum fasiculatum*). At the time of image collection, these weed species occurred at different growth stages, from cotyledon to about five true leaves.

**Table 1:** Various datasets used in the study.

	Image dataset name	Acquisition date	Crop/growth stage	Weed composition/growth stage	Weed density (plants m^−2^)	Image acquisition conditions	Train/Val/Test [images, annotations]	Annotation composition^a^ [MG, Grass, and Other]
1	Cotton 1 (Test data referred to as Cot1)	May 06, 2020	Cotton: 4–5 leaves	MG: cotyledon-4 leaves JG: 2–3 leaves TM: 2–3 leaves	18	Sunny	Train: [460, 8,580] Val: [100, 721] Test: [100, 848]	[19.3, 79.5, 1.2] [22.4, 74.2, 3.4] [51.8, 48.1, 1.1]
2	Cotton 2 (referred to as Cot2)	June 13, 2021	Cotton: 2–4 leaves	MG: cotyledon-6 leaves TM: 2–4 leaves	21	Partially cloudy	Test: [95, 600]	[36, 63.8, 0.2]
3	Soybean 1 (Test data referred to as Soy1)	May 06, 2020	Soybean: 6–7 leaves	MG: cotyledon-4 leaves JG: 2–3 leaves TM: 2–3 leaves	17	Sunny	Train: [115, 990] Val: [25, 200] Test: [100, 848]	[46.4, 53.48, 0.07] [48.4, 50.8, 0.8] [54.22, 43.22, 2.56]
4	Soybean 2 (referred to as Soy2)	May 14, 2021	Soybean: 1–3 leaves	MG: cotyledon-6 leaves TM: 2–4 true leaves	21	Cloudy	Test: [97, 547]	[63.07, 35.4, 1.53]
5	Corn 1 (Test data referred to as Corn1)	May 07, 2021	Corn: 2–3 leaves	MG: cotyledon-3 leaves JG: 2–3 leaves TM: 2–3 leaves	18	Sunny	Train: [115, 1,010] Val: [25, 215] Test: [100, 890]	[81.16, 16.75, 2.1] [95.2, 4.1, 0.7] [94.62, 4.9, 0.48]
6	Corn 2 (referred to as Corn2)	May 14, 2021	Corn: 3–4 leaves	MG: cotyledon-6 leaves TM: 2–4 true leaves	23	Cloudy	Test: [95, 559]	[80.5, 17.5, 2]

Train, training; Val, validation; MG, morningglories; TM, texas millet; and JG, johnsongrass.

a The annotations statistics shown within the brackets are given in %.

### Workflow

**Figure 2:**
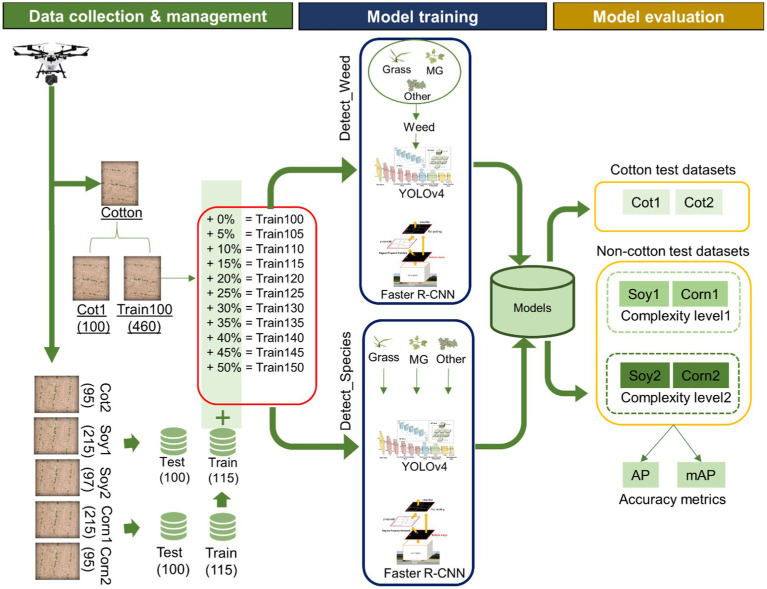
Schematic showing the workflow used in the study. The study began with data collection using an UAV and the collected data were distributed for training and test datasets. Data management was followed by model training under two detection schemes: Detect_Weed (detecting at weed/crop level) and Detect_Species (detecting at weed species level). After the models were trained, they were evaluated on the test datasets (Other was excluded during the calculation of accuracy metrics). Average Precision (AP) and Mean Average Precision (mAP) was used as the metrics for performance evaluation.

The methodological workflow for this study involved three major steps: Data collection and management, model training, and model performance evaluation on different test datasets. See Figure 2 for a schematic diagram showing the workflow followed in this research. The following sections describe these three steps in more detail.

#### High-Resolution Digital Image Collection

A 100-megapixel FUJIFILM GFX100 medium format mirrorless RGB imaging camera was integrated with a multi-copter drone, Hylio AG-110 (Hylio Inc., TX, United States) to capture high-resolution aerial images of the crop fields (Figure 1). The images were captured by the drone operating at 4.9 m aboveground level and a speed of 0.61 m/s. The FUJIFILM GF 32–64 mm f/4 R LM WR lens was set at a focal length of 64 mm, shutter speed at 1/4,000 s, ISO at 1250, and f-stop at 8, which resulted in high-quality images with a spatial resolution of 0.274 mm/pixel at the given flying height. Under such configurations, image resolution and quality were sufficient for young grass seedlings to be recognized in the images. However, the wind thrust (i.e., downwash) from the drone operation impacted some plants, causing them to look unreal in the images. They were excluded from the dataset before further analysis. All the images were stored in standard PNG format at 16-bit depth. Table 1 describes the details of the different image datasets collected in the study.

A total of three flights were made to capture images for all the crops in 2020 and 2021. Two image datasets for each crop (Cotton 1 & Cotton 2, Soybean 1 & Soybean 2, and Corn 1 & Corn 2) were acquired (Table 1). For each crop, the second image dataset (e.g., Cotton 2) differed from the first dataset with respect to crop growth stage, weed density, and image acquisition conditions. Cotton 1 was the prime dataset for this study as this consisted of cotton-weed images that were used for building the main model. This dataset was split into training (hereafter referred to as “Train100”), validation, and test datasets. Soybean 1 and Corn 1 datasets were also partitioned similarly to supplement training and validation images to Train100 during cross-applicability improvements later on. All images in Cotton 2, Soybean 2, and Corn 2 were used for testing purposes. Hereafter, these test datasets are referred to as “Cot1,” “Cot2,” “Soy1,” “Soy2,” “Corn1,” and “Corn2” for respective crops.

#### Weed Detection

##### Image Annotations

For this study, the images were annotated and recorded in COCO format as this format is inter-changeable to several formats quickly and easily. The VGG image annotator (Dutta and Zisserman, 2019) was used to annotate the weeds with bounding boxes in each image. The annotations were recorded for three categories: morningglories (MG), grasses (Grass), and other weed species (Other). Both Texas millet and johnsongrass seedlings were labeled as “Grass” during annotation as classifying them was not the scope of this study.

##### Weed Detection in Cotton

With respect to the first objective, i.e., develop and evaluate models for weed detection in cotton, the detection frameworks were trained with Train100. Train100 comprised of 8,580 annotations altogether, out of which MG, Grass, and Other represented 19.3, 79.5, and 1.2%, respectively (Table 2). Two popular object detection frameworks, YOLOv4 and Faster R-CNN, were used in this study. YOLOv4 is the 4th subsequent version of the YOLO (Redmon et al., 2016), developed recently by Bochkovskiy et al. (2020). This framework is a one-stage object detector that divides images into several grids and calculates the probabilities that the cell grids belong to a certain class by computing several feature maps. The bounding boxes are then predicted based on grids with the highest probability for the respective classes. The detector sees the entire image during training and inferences for encoding contextual information about classes. Faster R-CNN is the subsequent version of Fast R-CNN (Girshick, 2015) developed by Ren et al. (2017). In contrast to the YOLO frameworks, Faster R-CNN is a two-stage object detector composed of two modules working together. The first module is a Region Proposal Network (RPN) that proposes several candidate regions in the image. The second module is the detector that first extracts features from dense feature maps for the regions selected during RPN and then calculates the confidence score for each region that contains the object of interest (Girshick, 2015).

**Table 2:** Various training datasets evaluated in the study for training YOLOv4 and Faster R-CNN and annotations record for each training dataset.

Training dataset	Non-cotton images (%)^a^	Annotations			
		MG (%)	Grass (%)	Other (%)	Total
Train100^b^	0	19.3	79.5	1.20	8,580
Train105	5	20.0	78.8	1.25	8,775
Train110	10	20.7	78.0	1.23	8,915
Train115	15	21.7	77.1	1.21	9,072
Train120	20	22.9	75.9	1.19	9,234
Train125	25	23.0	75.7	1.33	9,480
Train130	30	23.9	74.7	1.32	9,689
Train135	35	24.3	74.4	1.34	9,827
Train140	40	25.0	73.7	1.32	9,970
Train145	45	25.5	73.2	1.31	10,113
Train150	50	25.7	73.0	1.29	10,198

MG-Morningglories; Grass-Grass weeds; and Other-Weeds other than MG and Grass.

a The numerical figures in this column indicate the percentage of images added to Train100 (i.e., 460 images).

b Train100 had a total of 460 cotton images and 0 non-cotton images.

“Train100” represents the dataset with cotton images only, i.e., no non-cotton images. The last two digits of training dataset names represent the percentage of non-cotton images added to Train100 randomly for building the respective training dataset. The percentage was with respect to Train100.

On-the-fly augmentation of data was carried out for both the frameworks. The “mosaic” augmentation (Bochkovskiy et al., 2020) was enabled for YOLOv4, whereas the “flip and resize” augmentation was performed with the default data loader when training Faster R-CNN. Pre-trained models as provided by the github sources (https://github.com/facebookresearch/detectron2 for Faster R-CNN and https://github.com/AlexeyAB/darknet for YOLOV4 for YOLOv4) were used for model initialization. A mini-batch Stochastic Gradient Descent method was used for model loss optimization for both frameworks. Faster R-CNN was trained for 50,000 iterations whereas YOLOv4 was trained for 6,000 epochs. The definition for *iterations* and *epochs* for these frameworks implies different meanings and are explained in their respective github documentation resource. The model weights were saved after every certain number of iterations or epochs so that the weight resulting in the highest validation accuracy can be chosen at the end for further analysis. Because of the differences in their detection mechanisms, these two frameworks could provide different results for the same detection problem. Hence, evaluation of these two frameworks can provide valuable insights into what level of accuracy can be expected for the given detection problem.

Hereafter, the model trained with Train100 is referred to as the “main cotton model.” Two different schemes were designed for weed detection. In the first scheme, hereafter referred to as “Detect_Weed,” frameworks were trained to detect weeds at the meta-level irrespective of the species. The label names for MG, Grass, and Other were merged and labeled as “Weed” while training under this scheme. However, in the second scheme, hereafter referred to as “Detect_Species,” frameworks were trained to detect weeds at the species level. For training this scheme, the original annotation dataset that had separate labels for MG, Grass, and Other were used. These schemes have different significance depending on how they are utilized for management. Currently, most of the mechanical platforms for real-time weed control employ “Detect_Weed” scheme for precision control actions (Gai et al., 2020). In most of the existing commercial platforms, detectors are trained to only detect weeds, but not required to classify them at the species level, as the weeds are pulled, zapped, or clipped regardless of species in these platforms. However, selective herbicide spray systems would require detection and classification of individual weeds for species-specific herbicide input. Hence, it may be informative to investigate how these two frameworks behave under these weed detection schemes.

##### Cross-Crop Applicability Analysis

With respect to the second objective, i.e., assess the scope and prospects for applying the main cotton models to corn and soybean, the performance of the main cotton models was evaluated for each test dataset. In addition, the four non-cotton test datasets (i.e., Soy1, Soy2, Corn1, and Corn2) were grouped into two complexity levels based on their similarity in weed pressure conditions and image acquisition environment. It was assumed that these factors would have more influence than the similarity between crops. Thus, Soy1 and Corn1 were grouped under complexity level 1, while Soy2, and Corn2 under level 2. Cot2 was not grouped under any complexity level, but was rather considered as a replicate of Cot1. In the complexity level 1, the Soy1 and Corn1 differed from the Cotton 1 dataset only for the background crop species, whereas the weed density, growth stages of weeds, and image acquisition conditions were similar. In the complexity level 2, the datasets differed not only for the background crop species, but also for weed density, growth stages of weeds, and light conditions; these differences constitute a higher level of complexity to the weed detection process. Evaluations with these two complexity levels advance our understanding of the model performances under various environments.

##### Cross-Crop Applicability Improvement With Training Size Expansion

The third objective was to test if supplementing Train100 with additional training images from Soybean 1 and Corn 1 image datasets improves prediction for corn and soybean. As the frameworks were trained to recognize only the weeds and consider crops as part of the background, changes in crop species might confuse the frameworks as to what comprises the background. This confusion intensifies when the frameworks infer upon crop species that were never seen before. Due to this situation, it was assumed that exposing these unseen crops to the frameworks might help boost the confidence score for background. It was more desirable to achieve considerable improvement in the performance with a minimal number of Soybean 1 and Corn 1 images. For this purpose, 10 additional training datasets were prepared by randomly selecting an equal proportion of soybean and corn images and adding them to the main train dataset (i.e., Train100) such that the new dataset size did not exceed 150% of the Train100 size (Table 2). Both frameworks were trained independently using 10 different training datasets listed in Table 2 under the two detection schemes and were validated against test datasets. The same pre-trained models provided by the github source were used for model initialization for each training dataset. Moreover, configurations were also kept the same across training datasets for these two frameworks.

#### Accuracy Metrics for Performance Evaluation

The standard performance metric called Mean Average Precision (mAP) was calculated to assess the performance of weed detection under Detect_Species, whereas Average Precision (AP) was used as the performance metric for Detect_Weed. In recent years, these metrics have been frequently used to assess the accuracy of object detection tasks. mAP is a mean of AP calculated for each class to be detected/predicted by the model. AP for each class is calculated as the area under a precision-recall curve. The area is determined in two stages. First, the recall values are evenly segmented to 11 parts starting from 0 to 1. Second, the maximum precision value is measured at each level of recall and averaged to determine AP (Eq. 1).


(1) $$AP=\frac{1}{11}\underset{r\in \left\{0,0.1,0.2\ldots 1\right\}}{\sum }p_{\text{max}}\left(r\right)$$

where p_(max)_ represents maximum precision measured at respective recall (r) level.

Precision and recall values are in turn calculated using the Eqs 2, 3, respectively.


(2) $$\mathrm{Precision}=\frac{TP}{TP+FP}$$

(3) $$\mathrm{Recall}=\frac{TP}{TP+FN}$$

where *TP*, *FP*, and *FN* denote true positive, false positive, and false negative samples, respectively.

True positives, false positives, and false negatives are identified with the help of the Intersection over Union (IoU) ratio. This ratio is calculated by comparing the ground truth box with the model predicted box. If the ratio is above the user-defined threshold, the predicted box is labeled as TP. In this study, the threshold for IoU was set to 0.5. The mAP value ranges between 0 and 1, with 0 indicating null accuracy and 1 indicating perfect accuracy. Only the AP for MG and Grass were averaged to calculate mAP under Detect_Species. AP for Other were found to be very low due to a very small test sample size during the evaluation which led to non-representative mAP values; thus, the accuracy for Other category was excluded during the evaluation process for both frameworks and schemes.

## Results and Discussion

### Performance of the Main Cotton Model Over Cotton Test Datasets

Two popular object detection frameworks, YOLOv4 and Faster R-CNN were trained to detect weeds in cotton and non-cotton crops. Train100 was used to build two cotton-weed detection models under different detection schemes for each framework. Both YOLOv4 and Faster R-CNN provided reasonably fair accuracy levels under both detection schemes for Cot1 (Table 3). Under Detect_Species, AP was higher for MG compared to Grass. Although grasses were visible to naked eyes and also discernible in the images, the model failed to detect a few grass instances. On the contrary, the model led to over-detection (i.e., more plants were predicted than what was present) when these grasses had multiple tillers spread out. Lottes et al. (2018) also observed lower AP for grasses compared to broadleaves when they tested their weed detection model on UAV imageries. However, the opposite was true when they tested on images collected using a ground robot.

**Table 3:** Accuracy obtained for various test datasets with YOLOv4 and Faster R-CNN under Detect_Weed and Detect_Species using the main cotton model.

	Detect_Weed		Detect_Species					
	YOLOv4	Faster R-CNN	YOLOv4			Faster R-CNN		
	AP	AP	AP (MG)	AP (Grass)	mAP	AP (MG)	AP (Grass)	mAP
Cot1	0.88	0.87	0.88	0.83	0.85	0.86	0.79	0.83
Cot2	0.79	0.74	0.71	0.79	0.75	0.60	0.70	0.65
Soy1	0.83	0.76	0.83	0.75	0.79	0.72	0.70	0.71
Soy2	0.35	0.60	0.63	0.64	0.64	0.72	0.49	0.61
Corn1	0.72	0.62	0.88	0.15	0.52	0.78	0.15	0.47
Corn2	0.33	0.39	0.65	0.15	0.40	0.54	0.03	0.29

MG-Morningglories; Grass-Grass weeds; AP, average precision; and mAP, mean average precision. AP and mAP values were computed to assess the performance of the main cotton model over the test datasets. mAP was calculated by averaging AP for MG and Grass. AP was calculated as a function of precision and recall values obtained when Intersection Over Union (IoU) was set to 0.5.

When the same models were tested over the second cotton dataset (i.e., Cot2) collected in 2021, the AP & mAP values declined by 12.5 & 14.5% and 11.7 & 22.5% for YOLOv4 and Faster R-CNN, respectively. Unlike Cot1, AP was higher for Grass than for MG for both frameworks under Detect_Species. It should be noted that Cot2 differed from Cot1 in three aspects: (1) Cot2 had a relatively higher density of weeds and the median size of MG and Grass differed from that of Cot1, (2) some of the cotton plants in Cot2 had slightly different visual appearance due to herbicide drift, and (3) the illumination conditions for Cot2 was slightly darker than that of Cot1. Hu et al. (2021) suggested that illumination conditions can affect weed detection accuracy. With respect to herbicide drift impact, Suarez et al. (2017) found in cotton that drift can lead to a significant change in the spectral behavior of the crop. All these reports indicate that morphological, agronomical, and illumination differences can be attributed to the lower accuracy levels observed for Cot2.

Very few studies have looked at weed detection and mapping in cotton. Alchanatis et al. (2005) used rank order algorithms and neighborhood operations to detect broadleaves and grass weeds in cotton. With their approach, 86% of the true weed area was correctly identified, with only 14% misclassified as cotton. Lamm et al. (2002) developed an early growth stage weed control system for cotton. Using morphological analysis such as binarization and erosion, their system was able to correctly identify and spray 88.8% of the weeds. On a different note, both frameworks used in this study have been already used in other weed detection studies. For example, Gao et al. (2020) employed YOLOv4 and Tiny YOLO to detect field bindweed (*Convolvulus sepium*) in sugar beet (*Beta vulgaris*) fields. They used synthetic images in addition to real images to train the framework and obtained an mAP_50_ value of 0.829 for field bindweed detection. Osorio et al. (2020) used YOLOv3 and other object detection frameworks for weed detection in commercial lettuce crops and obtained an overall accuracy of 89% with YOLOv3. Using the Faster R-CNN framework with the Inception_ResNet-V2 backbone, Le et al. (2020) achieved an mAP_0.50_ value of 0.55 for detection of wild radish (*Raphanus raphanistrum*) and capeweed (*Arctotheca calendula*) in barley. The overall accuracy obtained in this study for weed detection compares well with reported accuracies by past studies.

### Cross-Crop Applicability of Main Cotton Models

**Figure 3:**
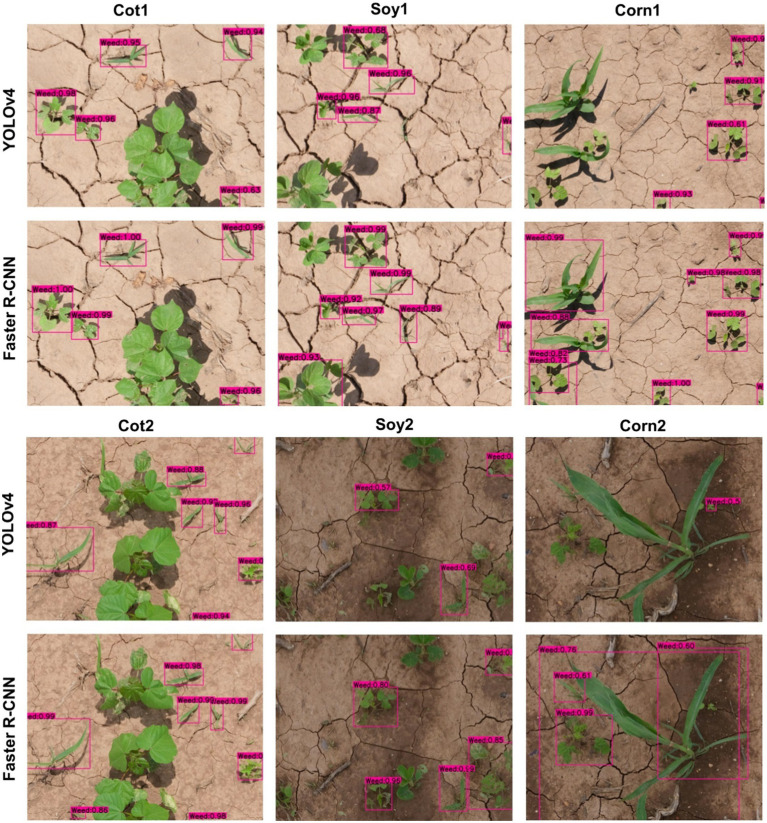
Weed detection using bounding boxes by the main cotton models under “Detect_Weed” scheme for various test datasets used in the study. YOLOv4 and Faster R-convolutional neural network (CNN) were trained with the Train100 dataset (i.e., dataset containing cotton images only) to develop the main cotton models. Under this scheme, MG, Grass, and Other were combined into “Weed” category while training the model.

**Figure 4:**
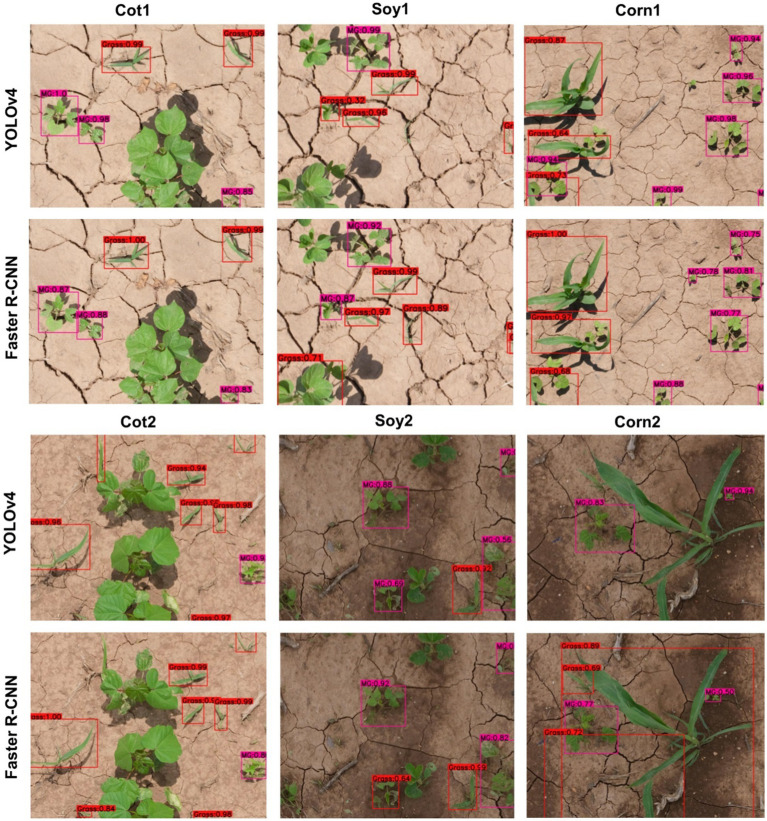
Bounding boxes generated for MG and Grass by the main cotton models under “Detect_Species” scheme for various test datasets used in the study. YOLOv4 and Faster R-CNN were trained with the Train100 dataset (i.e., dataset containing cotton images only) to develop the main cotton models. Under this scheme, MG, Grass, and Other were trained as separate categories.

**Figure 5:**
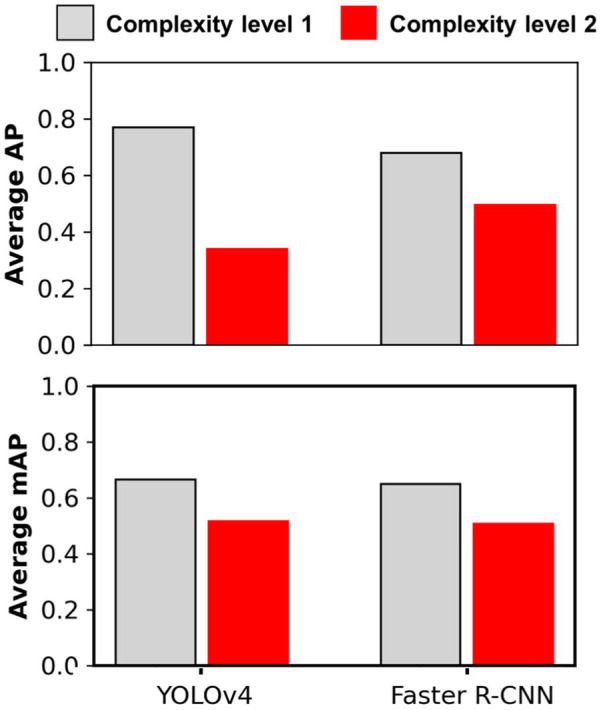
Average Precision and mAP achieved for different complexity level datasets with main cotton models. Complexity level 1 datasets include Soy1 and Corn1, whereas level 2 include Soy2 and Corn2. The main cotton models were derived by training the detection frameworks (YOLOv4 and Faster R-CNN) with Train100 (i.e., dataset containing cotton images only). The AP/mAP for datasets under each complexity level were averaged to derive average AP and mAP.

The main cotton models were also applied over non-cotton test datasets (i.e., Soy1, Soy2, Corn1, and Corn2) under both detection schemes. The main goal was to see if one crop-based weed detection model can be used to detect the same weeds in other crop species under similar or different agronomic and image acquisition conditions. The detection results by both frameworks for different test datasets under Detect_Weed and Detect_Species are shown in Figures 3, 4, respectively for qualitative evaluation. The Detect_Species cotton model performed satisfactorily for Soy1 and Soy2 datasets, while not so effectively for Corn1 and Corn 2 datasets. The Detect_Weed model performed the same way except that AP was higher for Corn1 but not for Soy2. The significant difference in performance between Faster R-CNN and YOLOv4 for Soy2 under Detect_Weed is notable. In this regard, YOLOv4 predictions on Soy2 images were further investigated. Several MG were not detected by the model, resulting in many false negatives. AP/mAP for non-cotton test datasets was not better than that of Cot1 for both frameworks. Among non-cotton test datasets, the highest AP/mAP was obtained for Soy1 for both frameworks (Table 3). Further, in general, the model performed relatively better on complexity level 1 than level 2 (Figure 5). The difference in performance was more obvious under Detect_Weed for both frameworks.

It was notable that Soy1 yielded higher AP/mAP values than Cot2 for both frameworks under both schemes. The authors could think of two reasons for this outcome: Soy1 had similar weed density and sizes to that of Train100; further, Soy1 and Train100 datasets were acquired at the same time, and hence illumination conditions were exactly the same. Here, higher accuracy for Soy1 suggests that illumination conditions and weed density can impose more influence on the detection accuracy. In general, higher accuracies were obtained for soybean datasets compared to corn datasets. The main reason could be the confusion between Grass and corn plants. A few instances of corn plants were detected as Grass by the model as they looked similar during early growth stages. Such misclassification was also observed when corn was distinctively larger than grasses. This suggests that the model may have focused more on the canopy structure than canopy size. Further, the detection performances between complexity levels were in line with our expectations. The primary reason for higher accuracy with complexity level 1 was the similar illumination conditions and weed density to the training dataset, i.e., Train100 as compared to the level 2 test datasets.

### Cross-Crop Applicability Improvement With Additional Non-cotton Image Datasets

**Figure 6:**
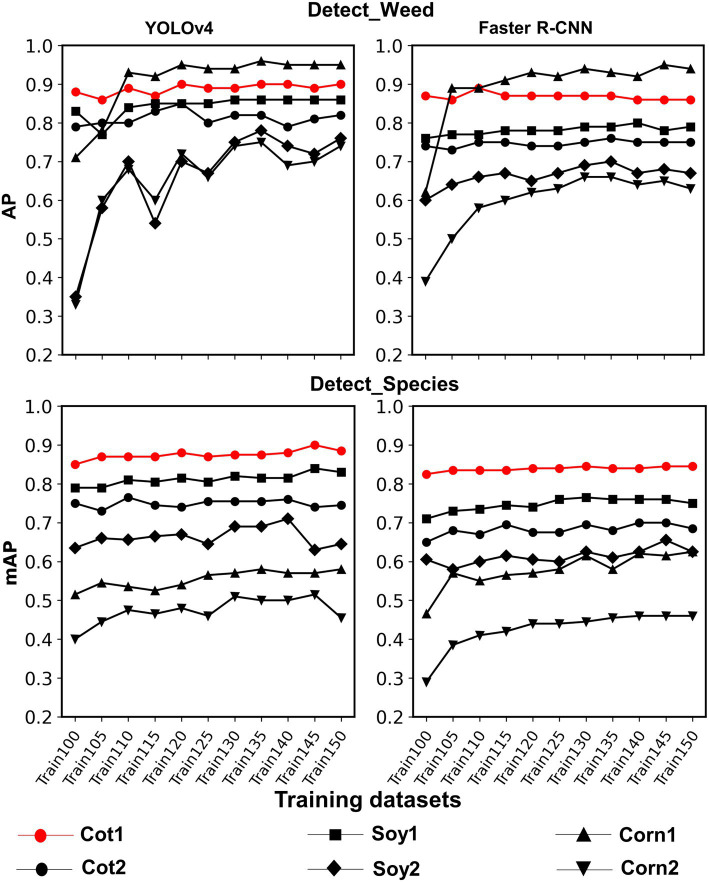
Line plots showing AP and mAP achieved with various training datasets for each test dataset used in the study for both frameworks and detection schemes. Various training datasets were created by adding Soybean 1 and Corn 1 training images to the original dataset, i.e., Train100. These non-cotton crop images were added 5% at a time until they amounted to 50% of Train100. The last two digits in the training dataset name denote the % of images added to Train100.

**Figure 7:**
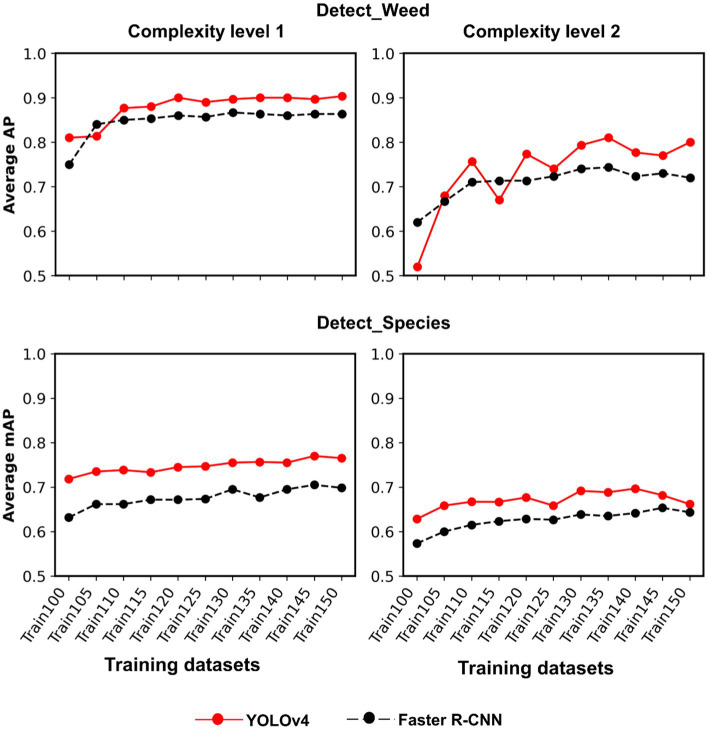
Line plots showing AP and mAP achieved for each complexity level with YOLOv4 and Faster R-CNN. Complexity level 1 datasets include Soy1 and Corn1, whereas level 2 include Soy2 and Corn2. AP and mAP for Cot1 dataset were also included in the averaging process of each complexity level to understand how well the models perform with both cotton and non-cotton datasets.

Train100 was supplemented with different amounts of training images from Soy1 and Corn1 to generate various training datasets. These datasets were used to train new models under two detection schemes and finally, the built models were tested over cotton and non-cotton test datasets. Both frameworks showed general increments in accuracy with the addition of non-cotton crop images under both detection schemes (Figure 6). The rate of increment, however, varied across test datasets, frameworks, and detection schemes (Table 4). The trend was relatively smoother for Faster R-CNN compared to YOLOv4 for all test datasets. The increment was the highest for Corn2 and the lowest for either of the cotton test datasets for both frameworks and detection schemes. AP/mAP for test datasets under each complexity level were averaged along with Cot1 values to calculate average AP/mAP (Figure 7). The trend was smoother for Faster R-CNN compared to YOLOv4 for all complexity levels.

**Table 4:** The maximum rate of increment in accuracy for various test datasets with the addition of non-cotton images.

	Detect_Weed (AP%)		Detect_Species (mAP%)	
	YOLOv4	Faster R-CNN	YOLOv4	Faster R-CNN
Cot1	2.27	2.29	5.89	2.42
Cot2	7.60	2.70	2.00	7.70
Soy1	3.61	5.26	6.32	7.74
Soy2	122.8	16.00	11.90	8.27
Corn1	31.9	53.22	12.62	34.40
Corn2	127.27	69.23	28.75	58.62

AP, average precision; mAP, mean average precision. The rate was determined by subtracting the accuracy obtained with Train100 (no non-cotton images) from the highest accuracy obtained among all training datasets for the respective test dataset.

### Scope and Limitations of the Study

Cross-crop applicability assessments conducted in this study provides useful insights into how models can be generalized for broad application. Such generalization could save enormous efforts and resources and help make rapid progress toward effective site-specific weed management. Cross-applicability has become an absolute necessity owing to the huge data requirements by the CNN models for a given crop-weed environment. Often, a significant amount of data resources is used to train a weed detection model for a single crop environment. For example, Yu et al. (2019) used a total of 29,000 images to train a model that could detect multiple weeds in perennial ryegrass. Czymmek et al. (2019) trained a model to detect weeds in organic carrot farms using 2,500 images. It is increasingly important to focus on how these data resources can be exploited strategically for maximizing efficiency and productivity. By testing the approach of data supplementation, this study demonstrated that cross-crop applicability can be improved with such tactics.

It should be noted that this study evaluated CNN model cross-applicability for crops that had similar weed compositions. The cross-crop applicability findings from this study do not apply to crops differing in weed species composition. In other words, the models would fail to perform if applied over soybean and corn infested with other weed species. A single crop-based model may not be effectively applied at regional scales where weed composition differs. Furthermore, not all the hyperparameters for both frameworks used in the study were tuned, but rather used as defaults in the settings. The reported accuracies may change if parameters are tuned.

## Conclusion

The study explored two popular object detection frameworks under two useful detection schemes for weed detection in cotton. The study also evaluated the feasibility of cross-crop applicability of the cotton model and experimented with several amounts of non-cotton images to improve cross-applicability. Based on the results, the following main conclusions could be derived:

- The cotton model achieved reasonably high weed detection accuracy for cotton test datasets.
- The cotton model achieved a fair level of accuracy for non-cotton crops infested with similar weed compositions. On average, the performance was better for soybean than for corn.
- The cross-crop applicability was improved (AP/mAP: +3.61 to 127.27%) when Train100 was supplemented with non-cotton images.

The outcomes of this study are expected to advance our understanding of cross-crop applicability of weed detection models. Such understanding will guide our efforts toward optimal use of data resources and accelerate weed detection, mapping, and site-specific management in agricultural systems. In the future, CNN model cross-applicability will be assessed for additional crops and different levels of complexities.

## Data Availability Statement

The raw data supporting the conclusions of this article will be made available by the authors, without undue reservation.

## Author Contributions

MB and BS: conceptualization and experimental design. MB: funding acquisition, supervision, and project management. BS and CH: field data acquisition and analysis. BS: writing the first draft of the paper. MB, BS, and CH: paper editing and revisions. All authors contributed to the article and approved the submitted version.

## Funding

This study was funded in part by the USDA-Natural Resources Conservation Service-Conservation Innovation Grant (NRCS-CIG) program (award #NR213A750013G017) and Cotton Incorporated (award #20-739).

## Conflict of Interest

The authors declare that the research was conducted in the absence of any commercial or financial relationships that could be construed as a potential conflict of interest.

## Publisher’s Note

All claims expressed in this article are solely those of the authors and do not necessarily represent those of their affiliated organizations, or those of the publisher, the editors and the reviewers. Any product that may be evaluated in this article, or claim that may be made by its manufacturer, is not guaranteed or endorsed by the publisher.
